# Genetic Variation and Population Differentiation in a Medical Herb *Houttuynia cordata* in China Revealed by Inter-Simple Sequence Repeats (ISSRs)

**DOI:** 10.3390/ijms13078159

**Published:** 2012-07-02

**Authors:** Lin Wei, Xian-Jin Wu

**Affiliations:** 1 The Department of Life Science, Huaihua University, Huaihua 418008, China; E-Mail: hhweilin@163.com; 2 Key Laboratory of Hunan Province for Study and Utilization of Ethnic Medicinal Plant Resources, Huaihua 418008, China; 3 Key Laboratory of Hunan Higher Education for Hunan-Western Medicinal Plant and Ethnobotany, Huaihua 418008, China

**Keywords:** genetic variation, *Houttuynia cordata*, ISSR, population differentiation

## Abstract

*Houttuynia cordata* is an important traditional Chinese herb with unresolved genetics and taxonomy, which lead to potential problems in the conservation and utilization of the resource. Inter-simple sequence repeat (ISSR) markers were used to assess the level and distribution of genetic diversity in 226 individuals from 15 populations of *H. cordata* in China. ISSR analysis revealed low genetic variations within populations but high genetic differentiations among populations. This genetic structure probably mainly reflects the historical association among populations. Genetic cluster analysis showed that the basal clade is composed of populations from Southwest China, and the other populations have continuous and eastward distributions. The structure of genetic diversity in *H. cordata* demonstrated that this species might have survived in Southwest China during the glacial age, and subsequently experienced an eastern postglacial expansion. Based on the results of genetic analysis, it was proposed that as many as possible targeted populations for conservation be included.

## 1. Introduction

Saururaceae, a member of paleoherbs, is an ancient family with six species in four genera, *i.e*., *Anemopsis*, *Gymnotheca*, *Houttuynia* and *Saururus* [1]. *H. cordata* Thunb is the only species in the genus *Houttuynia* [2,3]. This herb propagates by formation and separation of underground stems and by parthenogenesis [4,5], although sexual reproduction has not yet been determined. It is distributed mainly in the central, southeastern and southwestern regions of China, and extends to Japan, Korea and Southeast Asia, where it grows in moist, shady places. *H. cordata* is used as a traditional Chinese medical herb. It plays a unique role in improving the immune system of patients with severe acute respiratory syndrome (SARS) [6], and the steam distillate from fresh plants inhibits herpes simplex virus type 1, influenza virus and human immunodeficiency virus type 1 without cytotoxicity [7]. *H. cordata* is also consumed as a vegetable in China for its special aroma. Although *H. cordata* is of high medicinal and economic value, wild *H. cordata* population resources are limited, which is a main hurdle for the breeding of new cultivated varieties. Therefore, it is critical to arrest this dearth of information on this important natural resource and study the genetic diversity of the remaining population.

Knowledge of population genetic structure provides evolutionary perspectives of a species, allows for the prediction of population response to natural and artificial changes in the future [8], and is essential for the successful preservation and utilization of rare species [9]. Wu *et al*. [10,11] have determined the geographic distribution of *H. cordata*, as well as the relationship between its genetic variation and life history based on morphological features, isozyme, chromosome number and RAMP markers. However, samples in these studies were collected from limited areas in the Sichuan Basin of China, and the genetic information was not sufficient for the conservation of the rare but valuable species.

Because large numbers of polymorphic fragments can be produced by inter-simple sequence repeats (ISSR)-PCR at a relatively low cost [12,13], this technique has great potential in analyzing genetic variation below species level, particularly population structure and differentiation, including gene flow among populations and genetic variation within a population [14]. In this paper, we analyze the genetic structure of *H. cordata* based on ISSRs using a large number of individuals sampled from wide areas including central, southeastern and southwestern China. The purposes of this study are to address the following questions. What is the level of genetic diversity and the degree of differentiation among populations in *H. cordata* from its main range? What does the genetic structure imply for the evolutionary history? How can the species be conserved and used effectively based on the revealed genetic information?

## 2. Results

### 2.1. Genetic Diversity

The 10 selected primers amplified a total of 115 reproducible and distinct ISSR bands with a percentage of polymorphic markers of 97.39%. Among the 115 bands scored, 25 bands (21.7%) were found in 50–100% of samples, 27 bands (23.5%) were found in 30–49% of samples, and 63 bands (54.8%) were found in less than 30% of samples. A few population-specific bands were observed in the data set. Table 1 describes the polymorphism in this species revealed by ISSR in detail. The percentage of polymorphic loci (*P_p_*) was 30.49% on average, ranging from 12.17% to 42.61% at the population level. Nei’s genetic diversity (*H*) was estimated to be 0.0923 at the population level and 0.2820 at the species level, while Shannon indices (*I*) were 0.1413 and 0.4338 at these levels, respectively. Among the 15 populations, population KM exhibited the highest level of variability (*H* = 0.1494; *I* = 0.2239), while population LX exhibited the lowest level of variability (*H* = 0.0364; *I* = 0.0565).

### 2.2. Genetic Differentiation

Differentiation among populations was significant in this species. The coefficient of genetic differentiation among populations (G*_ST_*, estimated by partitioning of the total gene diversity) was 0.6728. The level of gene flow among populations (*Nm*) was estimated to be 0.2431, indicating that there was a low migration rate among populations. This finding was consistent with the type of genetic structure predicted by the Shannon’s diversity index analysis, which suggested that 67.42% of the total variation was partitioned among populations. Table 2 shows an estimate of Nei’s genetic identities (*G_I_*) and genetic distance (*G_D_*) for each pairwise comparison between two populations. Genetic identities between populations varied from 0.6936 to 0.9184 with a mean of 0.7764 ± 0.0437. Correlative analysis with SAS software showed that there was no significant correlation either between genetic differentiation and latitude (*p* = 0.9408), or between genetic differentiation and longitude (*p* = 0.3368). The AMOVA indicated that most (77%) of the molecular variation in *H. cordata* populations existed among populations, with lesser amounts within populations (23%). Permutation tests (based on 999 permutations) suggest that the overall ΦPT was significantly different from the null distribution (ΦPT = 0.769, *p* = 0.010) (Table 3), which indicates the differences among populations are significant.

**Figure 1:**
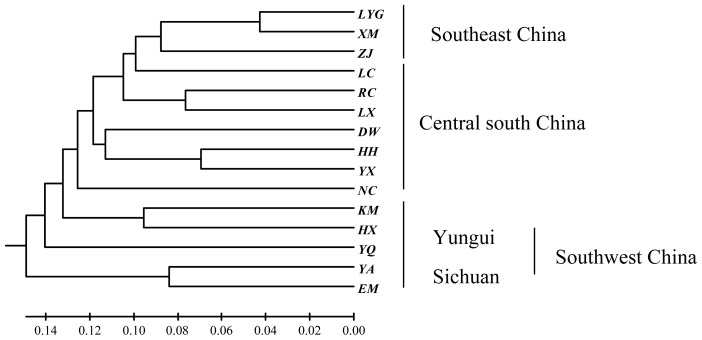
UPGMA dendrogram based on Nei’s (1972) genetic distance.

A UPGMA dendrogram based on Nei’s (1972) genetic distance indicated that the 15 populations were clustered into four geographically distinct groups, including Sichuan basin (EM, YA and YQ), Yungui Plateau (KM and HX), central south China (HH, YX and DW) and southeast China (LX, LC, ZJ, XM and LYG) (Figure 1). The Mantel tests indicated that there was no significant associated relationship between genetic distance and geographic distance among populations in *H. cordata* (*r* = 0.0400, *p* = 0.3760).

## 3. Discussion

### 3.1. Genetic Structure in H. cordata

The genetic structure of plant populations reflects interactions amongst a range of different processes, including the long-term evolutionary history of the species (such as shifts in distribution, habitat fragmentation and population isolation), mutation, genetic drift, mating systems, as well as gene flow and selection [15]. In this study, the genetic structure of *H. cordata* was generally characterized by high genetic variation at species level (Shannon indices of 0.4338 and Nei’s gene diversity of 0.2820) and low genetic variation within population (0.1413 and 0.0923 on average, respectively). These results were similar to those obtained using other molecular markers (such as RAPD and SSR) previously in a much smaller sample size [16]. This herb survived and adapted as a result of agamic reproduction in the central and southern areas of the Northern Hemisphere during late tertiary to early quaternary [1]. At the glacial maxima, *H. cordata* was restricted to refugia with an endurable temperature, and such areas were expected to be small. One of the major genetic consequences of long-term habitation at a small population size is high levels of genetic drift [17,18]. This factor could be, at least partially, responsible for the low genetic variation within population and high genetic variation among populations.

The individuals in different regions were not from the same migratory routes derived from distinct sister groups that once lived in the refuge. In the present study, 15 sampled populations were collected from 13 provinces in China. Latitude and longitude of the areas vary from 21°12′ (N) to 34°36′ (N) and from 101°31′ (E) to 119°36′ (E), respectively. Different geographical distribution ranges, the intricate landform and the weather conditions of the sampled populations might have limited migration. In this study, the coefficient of genetic differentiation (*G_ST_*) of the species was 0.6728, the proportion of diversity among populations based on the Shannon indices was similarly high (67.42%); correspondingly, the estimate of gene flow (Nm) is low (0.2431); and the AMOVA test also showed that there was high genetic diversity among populations (77%) and low genetic diversity within the population (23%). *H. cordata* is a relic of its agamic race [1], and the plant is small and short. This constrains gene flow via seed and pollen dispersal, and thus the plant fails to reproduce extraneously in each highly isolated population. Cytological examinations revealed that *H. cordata* is a polyploidy complex with 36 to 126 chromosomes [19], which hinders gene exchanges among populations harboring different numbers of chromosomes. Therefore, these patterns of variation are due not to ongoing gene flow, but rather to historical association among populations. According to the geographical distribution and the affinities shown in the UPGMA dendrogram (Figure 1), *H. cordata* might have survived in Southwest China during the last Pleistocene glaciation. The route extended southeastward of Southwest China along the Yungui Plateau Range, where the present distribution pattern appeared. The strong differentiation among populations may be mainly due to subsequent genetic drift and natural selection. Genetic drift changes the distribution of genetic variation either by reducing variation within a population or by increasing the differentiation among populations [18]. Although the UPGMA dendrogram (Figure 1) indicated that the distribution of *H. cordata* in China took on an overall pattern of eastward expansion, the lack of significant correlation between genetic distance and geographical distance supports the hypothesis of historical relationships and the subsequent action of genetic drift accounts for current-day patterns of variation.

### 3.2. Implication for Taxonomy

A new species *H. emeiensis* Z.Y. Zhu and S. L. Zhang in the genus *Houttuynia* was collected at E’mei mountain in Sichuan province, China, and classified by Zhu and Zhang according to the color of the stem, the width and length of the inflorescence, and the number of flowers and bract [20]. However, in this study, Nei’s original measures of genetic distance (Table 2) and the UPGMA dendrogram (Figure 1) failed to provide support for their conclusions. The largest and the smallest genetic distances between EM and other populations was 0.3659 (KM) and 0.1679 (YA), respectively, with a mean of 0.2882 ± 0.0608. The overall correlation between genetic differentiation and geographical distance was weak. Cluster analysis showed strong correlations between EM and YA, YQ and NC, KM and HX, as well as RC and LX. EM was not classified as one group based on ISSR markers. Since *H. emeiensis* (EM) did not stand alone in these analyses, it is necessary to deliberate in classifying it as a new species.

### 3.3. Implication for Conservation

Our results also provide theory for further protection of the germplasm resources. The low levels of genetic diversity within a population and the low gene flow among populations point towards the possibility of the possession of unique genotypes in a single population that are not found in other populations. It is therefore imperative for conservation planners, when designing conservation strategies for wild populations of *H. cordata* in China, to ensure that as many as possible separate populations are targeted for conservation rather than a few selected populations. *Ex situ* conservation may also be appropriate, because the total genetic diversity in a population of *H. cordata* may be adequately captured in only a few plants from the wild, which would not be the case for species with high levels of genetic diversity within a population. It would be beneficial to find ways to strengthen the gene flow between populations to maintain the natural genetic variation of *H. cordata*. Considering the high genetic differentiation among the wild populations, preservation of only a few populations may not adequately protect the genetic variation within the species in central China. Therefore, several populations throughout the entire range of the species in the country should be considered for conservation.

## 4. Experimental Section

### 4.1. Sample Collection

**Figure 2:**
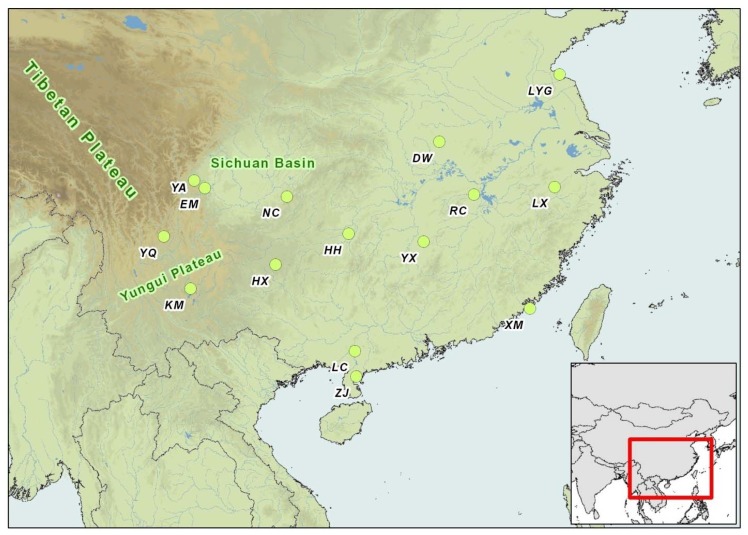
Locations of the sampled populations of *H. cordata*.

Leaf samples were collected from 266 individuals of *H. cordata* Thunb from 15 populations located in the natural distribution of the species in 13 provinces of China (Table 4 and Figure 2). The samples were taken from individuals at least 2 km apart in each population to avoid sampling clonal relatives. Leaves were dried, and preserved in silica gel until DNA extraction.

### 4.2. DNA Extraction, PCR Amplification and Electrophoresis

Genomic DNA was extracted from the leaves of each individual using CTAB protocol [21], dissolved in double distilled water, and quantified using a spectrophotometer comparing band intensities with known standards of lambda DNA on 1.5% agarose gels. In a preliminary experiment, 100 ISSR primers (designed by the University of British Columbia (UBC), Vancouver, Canada) were tested on five random individuals from all the populations. Ten primers (USB #807(AG)_8_T, #808(AG)_8_C, #810(GA)_8_, # 811(GA)_8_C, #812(GA)_8_A, #825(AC)_8_T, #826(AC)_8_C, #848(CA)_8_RG, #850(GT)_8_YC, and #873(GACA)_4_) that produced clear bands were chosen for all the 266 individuals in subsequent PCR reactions. For each primer analyzed, amplification was repeated once in the same situation to test the reproducibility of the ISSR bands, with a sample composed of two or three individuals selected randomly from each population.

PCR amplification was performed in a 10-μL reaction volume containing about 20 ng of template DNA, 1.0 μL 10× PCR buffer, 2.0–2.2 mM MgCl_2_, 0.21 mM dNTPs, 200 nM primers, 1.0 unit of Taq polymerase and double-distilled water. Amplification was performed in a PTC-100 thermal cycler (MJ Research, Inc.) programmed for an initial step of 3 min at 94 °C, followed by 35 cycles of 1 min at 94 °C, 1 min at 50 °C or 52 °C and 1.5 min at 72 °C, and a final 8 min extension at 72 °C. A negative control reaction without template DNA was also included to verify the absence of contamination. PCR products were separated in 1.2% agarose gels buffered with 1× TBE. Only bands reproducible in different PCR reactions were further analyzed. Bands with similar migration distance amplified from different individuals were considered homologous. A positive control (PCR products amplified from a standard individual) and a Gene Ruler DNA 100 bp ladder (Bio Basic Inc.) were run on each gel for homology assessments of PCR products on different gels.

### 4.3. Statistical Analyses

The presence or absence of a PCR band was scored as 1 or 0, respectively. Two people scored the bands together in order to minimize the scoring of artificial bands. Only bands that were consistently amplified were considered, while smearing and weak bands were excluded. We refrained from pruning the loci that fulfilled Lynch and Milligan’s [22] criterion because that might lead to significant bias in the estimation of population genetic parameters [23]. This bias was substantially eliminated if a high number of polymorphic dominant markers were generated [24]. Statistical analyses of ISSR patterns were based on the following assumptions: (1) ISSR fragments behave as diploid, and the dominant marker alleles are either present (amplified) or absent (non-amplified); (2) co-migrating fragments represent homologous loci; (3) polymorphic loci are inherited in a nuclear (Mendelian) fashion [25]; and (4) populations are in Hardy-Weinberg equilibrium (HWE; Fis = 0).

Four genetic diversity parameters were analyzed using POPGENE (version 1.31) [26]: effective number of alleles (*Ne*), Nei’s gene diversity (*H*) [27], Shannon’s Information index (*I*) [28], and percentage of polymorphic loci (*P_p_*). To examine population genetic structure, the proportion of genetic divergence was estimated within and among populations as described by Nei [27], and *G_ST_* was designated as the coefficient of gene differentiation among populations. The genetic identity (*G_I_*) and the genetic distance (*G_D_*) among populations were also computed using the model presented by Nei [17]. Gene flow estimates (*Nm*) were calculated as *Nm = (1* – *G_ST_)/4G_ST_* [29]. Differentiation among population was analyzed with an analysis of molecular variance (AMOVA) using Genalex 6.1 based on 999 permutations. The AMOVA procedure in Genalex follows the methods of Excoffier *et al.* 1992, which estimates and partitions total molecular variance within and between populations and then tests the significance of partitioned variance components using permutational testing procedures. When the data are binary, AMOVA calculates the ΦPT value, which is analogous to Fst representing the proportion of the total variance among populations [30–32].

An unweighted pair group method using an arithmetic average (UPGMA) dendrogram was performed on the data matrix of mean character difference between pairs of samples with software MEGA version 2.1 (downloaded from the website at http://www.oup-usa.org/sc/0195135857). The relationship between latitude and longitude and the molecular indices was calculated as a two-polynomial regression. Correlations were calculated with software SAS version 6.12.

Finally, a Mantel correlation test [33] was applied, by using GenAlEx 6, to a matrix of pairwise geographic and pairwise genetic distances, with 999 random permutations to determine significance.

## 5. Conclusions

In summary, ISSR analysis revealed low genetic variations within a population but high genetic differentiations among populations in the medical herb *H. cordata* in China. This genetic structure probably reflected the historical association among populations, and subsequent severe genetic drift of small and isolated populations further promoting random divergence among populations. The observed genetic structure of the populations implies that as many populations as possible should be considered for both *in situ* and *ex situ* conservation practices on this species.

## Figures and Tables

**Table 1:** Molecular variation measured as effective number of alleles (*N*e), Nei’s (1973) gene diversity (*H*), the Shannon index (*I*), and the percentage of polymorphic bands (*P*_p_) per population as well as their Standard deviations (S.D.), population-specific bands (PSB).

Pop. ID	*Ne* (S.D.)	*H* (S.D.)	*I* (S.D.)	*P_p_*	PSB
LYG	1.1981 (0.3149)	0.1202 (0.1755)	0.1846 (0.2562)	40.00%	1
XM	1.1274 (0.2552)	0.0802 (0.1475)	0.1265 (0.2191)	30.43%	0
YA	1.1069 (0.2486)	0.0653 (0.1417)	0.1011 (0.2088)	23.48%	0
EM	1.0741 (0.2290)	0.0427 (0.1253)	0.0639 (0.1816)	13.04%	0
KM	1.2556 (0.3574)	0.1494 (0.1940)	0.2239 (0.2798)	42.61%	1
HX	1.1263 (0.2690)	0.0767 (0.1515)	0.1178 (0.2229)	25.22%	0
NC	1.1460 (0.2913)	0.0871 (0.1618)	0.1325 (0.2362)	27.83%	0
LC	1.1491 (0.2757)	0.0924 (0.1586)	0.1438 (0.2336)	33.91%	3
DW	1.1964 (0.3439)	0.1125 (0.1829)	0.1683 (0.2628)	32.17%	0
YQ	1.1817 (0.3235)	0.1057 (0.1783)	0.1586 (0.2572)	33.04%	0
HH	1.1555 (0.2667)	0.0998 (0.1541)	0.1596 (0.2288)	41.74%	0
YX	1.1964 (0.3123)	0.1198 (0.1732)	0.1859 (0.2520)	42.61%	0
RC	1.1083 (0.2663)	0.0630 (0.1471)	0.0942 (0.2137)	18.26%	0
LX	1.0583 (0.1854)	0.0364 (0.1085)	0.0565 (0.1626)	12.17%	1
ZJ	1.2272 (0.3463)	0.1335 (0.1869)	0.2018 (0.2697)	40.87%	1
Total	1.4622 (0.3210)	0.2820 (0.1596)	0.4338 (0.2097)	97.39%	7

**Table 2:** Nei’s (1972) original measures of genetic identity (above diagonal) and genetic distance (below diagonal).

Pop. ID	LYG	XM	YA	EM	KM	HX	NC	LC	DW	YQ	HH	YX	RC	LX	ZJ
LYG	-	0.9184	0.7467	0.7708	0.8137	0.7494	0.8114	0.8273	0.8431	0.7826	0.7941	0.8273	0.8333	0.8563	0.8571
XM	0.0851	-	0.7421	0.7461	0.8168	0.7842	0.8117	0.8484	0.8178	0.7637	0.8075	0.8350	0.8153	0.7976	0.8223
YA	0.2921	0.2982	-	0.8454	0.6834	0.7421	0.7413	0.7252	0.7511	0.7213	0.7800	0.7946	0.6960	0.7486	0.7845
EM	0.2604	0.2929	0.1679	-	0.6936	0.6943	0.7143	0.7144	0.7856	0.7202	0.7914	0.8097	0.7190	0.7320	0.7756
KM	0.2062	0.2023	0.3807	0.3659	-	0.8264	0.7670	0.7511	0.7231	0.7683	0.7756	0.7645	0.7496	0.7393	0.8153
HX	0.2885	0.2431	0.2983	0.3649	0.1907	-	0.7871	0.8088	0.7795	0.7435	0.7633	0.7820	0.7232	0.7210	0.7497
NC	0.2090	0.2087	0.2994	0.3365	0.2653	0.2393	-	0.8041	0.7495	0.7168	0.7590	0.7851	0.7693	0.7142	0.8032
LC	0.1896	0.1644	0.3213	0.3364	0.2862	0.2122	0.2180	-	0.7589	0.7265	0.7590	0.8043	0.8045	0.8137	0.7854
DW	0.1707	0.2011	0.2862	0.2413	0.3242	0.2491	0.2883	0.2759	-	0.7454	0.7918	0.8045	0.7268	0.7425	0.7970
YQ	0.2452	0.2695	0.3268	0.3282	0.2636	0.2964	0.3330	0.3195	0.2938	-	0.7422	0.7736	0.7566	0.7433	0.8016
HH	0.2306	0.2138	0.2484	0.2340	0.2541	0.2701	0.2757	0.2757	0.2335	0.2981	-	0.8704	0.7383	0.7721	0.7946
YX	0.1896	0.1804	0.2300	0.2111	0.2685	0.2458	0.2420	0.2178	0.2176	0.2567	0.1388	-	0.7909	0.7682	0.8399
RC	0.1824	0.2042	0.3624	0.3299	0.2882	0.3240	0.2622	0.2176	0.3191	0.2789	0.3034	0.2346	-	0.8584	0.7746
LX	0.1551	0.2261	0.2896	0.3119	0.3020	0.3272	0.3367	0.2062	0.2978	0.2967	0.2586	0.2637	0.1527	-	0.7974
ZJ	0.1542	0.1957	0.2427	0.2541	0.2041	0.2881	0.2192	0.2416	0.2269	0.2211	0.2299	0.1744	0.2554	0.2264	-

**Table 3:** Analysis of molecular variances for 15 *H. cordata* populations.

Source of Variation	Degrees of Freedom	Sum of Squares	Mean Squares	Variance Components	% Total Variance
Among populations	14	51.150	3.654	0.203	77%
Within populations	251	15.316	0.061	0.061	23%
Total	265	66.466		0.264	100%

Stat	Value		*p* (rand >= data)		
ΦPT	0.769		0.010		

**Table 4:** Populations of *H. cordata* Thunb examined in the inter-simple sequence repeat (ISSR) analysis.

Code	Populations	Number of Samples	Latitude (N)	Longitude (E)	Altitude (M)
LYG	Lianyungang, JiangSu	18	34°36′	119°36′	20
XM	Xiamen, FuJian	19	24°13′	118°14′	18
YA	Ya’an, SiChuan	20	29° 54′	102°54′	2636
EM	Emei, SiChuan	18	29°34′	103°24′	2449
KM	Kunming, YunNan	20	25°06′	102°44′	1895
HX	Huaxi, GuiZhou	18	26°10′	106°37′	1112
NC	Nanchuan, ChongQing	17	29°11′	107°08′	468
LC	Luchuan, GuangXi	20	22°20′	110°15′	218
DW	Dawu, HuBei	20	31°37′	114°06′	389
YQ	YanQiao, SiChuan	20	27°25′	101°31′	1987
HH	Huaihua, HuNan	20	27°32′	109°57′	866
YX	Youxian, HuNan	20	27°11′	113°23′	452
RC	Ruichang, JiangXi	17	29°17′	115°41′	157
LX	Lanxi, ZheJiang	13	29°37′	119°22′	121
ZJ	Zhanjiang, GuangDong	6	21°12′	110°18′	24
